# Sir Thomas Spencer Wells

**Published:** 1897-03

**Authors:** 


### Obituary.

Sir Thomas Spencer Wells, Bart., F. R. C. S., died at Cap d'Antibes, near Cannes, January 31, 1891, in the seventy-ninth year of his age. He was the eldest son of Mr. William Wells, of St. Albans, and was born at Hertford, February 3, 1818. In 1839–40 he was at St. Thomas's hospital, in 1841 he became a member of the College of Surgeons, and soon entered the Royal Navy as assistant surgeon. He served in the naval hospital at Malta for six years, but finally, in 1853, settled in practice in the west end of London. In 1854 he was elected surgeon to the Samaritan free hospital. He served in the Crimean war, but returned to his hospital work at the Samaritan upon the cessation of hostilities. In April, 1854, Wells assisted Baker Brown at his eighth ovariotomy, at the Essex hospital, which was the first time that Wells had ever seen the operation.

In February, 1858, Sir Spencer made his first completed ovariotomy, and in June, 1880, the number of his ovariotomies had reached 1,000. He continued in active service at the Samaritan hospital for twenty years, resigning in 1878. During this time he published many interesting works and monographs largely devoted to the consideration of diseases of the ovaries and ovarian and uterine tumors, and his final work appeared in 1885, entitled Diagnosis and Surgical Treatment of Abdominal Tumors.

Many years ago Sir Spencer Wells purchased the David Garrick estate, at Golder's Hill, on the North side of London, consisting of forty acres of ground, that he developed into a beautiful country seat. The house, as remodeled, is as nearly a perfect home as one could wish, and all the surroundings are attractive. It is just three-quarters of an hour's ride from his country home to his consulting rooms, in Grosvernor street, Grosvenor square. Lady Wells, a daughter of the late Mr. James Wright Sydenham, died in 1886. Sir Spencer leaves six daughters, one of whom married, in 1889, Mr. Swinburne-Hanham. He is succeeded to the baronetcy by Arthur Spencer Wells, M. A., Cantab., his only son, who was private secretary to Sir William Vernon Harcourt when he filled the office of chancellor of the exchequer in the last Gladstone ministry.

Sir Spencer Wells was made president of the Royal College of Surgeons in 1882, and has received numerous honors from all parts of the civilised world. His body was cremated at Woking. It is safe to say that no man of the present century has left a more indelible stamp of his work on the surgery of the abdomen than Sir Thomas Spencer Wells.

